# *Kalanchoe brasiliensis* Cambess., a Promising Natural Source of Antioxidant and Antibiotic Agents against Multidrug-Resistant Pathogens for the Treatment of *Salmonella* Gastroenteritis

**DOI:** 10.1155/2019/9245951

**Published:** 2019-11-11

**Authors:** Oscar Alejandro Santos Mayorga, Ygor Ferreira Garcia da Costa, Jucélia Barbosa da Silva, Elita Scio, Adriana Lúcia Pires Ferreira, Orlando Vieira de Sousa, Maria Silvana Alves

**Affiliations:** ^1^Programa de Pós-Graduação em Ciências Farmacêuticas, Centro de Pesquisas Farmacêuticas, Faculdade de Farmácia, Universidade Federal de Juiz de Fora, Juiz de Fora, Minas Gerais CEP 36.036-900, Brazil; ^2^Laboratório de Bioatividade Celular e Molecular, Centro de Pesquisas Farmacêuticas, Faculdade de Farmácia, Universidade Federal de Juiz de Fora, Juiz de Fora, Minas Gerais CEP 36.036-900, Brazil; ^3^Laboratório de Produtos Naturais Bioativos, Departamento de Bioquímica, Instituto de Ciências Biológicas, Universidade Federal de Juiz de Fora, Juiz de Fora, Minas Gerais CEP 36.036-900, Brazil; ^4^Hospital Universitário Clementino Fraga Filho, Universidade Federal do Rio de Janeiro, Rio de Janeiro, Rio de Janeiro CEP 21.941-913, Brazil; ^5^Laboratório de Química Biomedicinal e Farmacologia Aplicada, Faculdade de Farmácia, Universidade Federal de Juiz de Fora, Juiz de Fora, Minas Gerais CEP 36.036-900, Brazil

## Abstract

*Kalanchoe brasiliensis* Cambess. is a native Brazilian plant popularly known as “saião”, and the juice of its fresh leaves is traditionally used to treat several disorders, including inflammatory and infectious processes such as dysentery. The goals of this study were to characterize the phytochemical composition and investigate the antioxidant activity, the antibiotic effect, and the mode of action against *Salmonella* of the hydroethanolic extracts from *K. brasiliensis* leaves collected in the summer and spring Brazilian seasons. These extracts had their chemical composition established by high-performance liquid chromatography with diode-array detection. Total phenolic and flavonoid contents were spectrophotometrically determined. 2,2-Diphenyl-1-picryl-hydrazyl radical scavenging, phosphomolybdenum reducing power and *β*-carotene bleaching assays were carried out to evaluate the antioxidant capacity. Antibiotic potential was assessed by minimal inhibitory concentration against 8 bacterial ATCC® and 5 methicillin-resistant *Staphylococcus aureus* and 5 *Salmonella* clinical strains. The mode of action was investigated by time-kill, bacterial cell viability, and leakage of compounds absorbing at 280 nm assays against *Salmonella*. Chromatographic profile and UV spectrum analyses suggested the significant presence of flavonoid type patuletin and eupafolin derivatives, and no difference between both periods of collection was noted. Significant amounts of total phenolic and flavonoid contents and a promising antioxidant capacity were observed. Hydroethanolic extracts (70%, summer and spring) were the most active against the tested Gram-positive and Gram-negative bacterial strains, showing the bacteriostatic action of 5000 *μ*g/mL. Time-kill data demonstrated that these extracts were able to reduce the *Salmonella* growth rate. Cell number was reduced with release of the bacterial content. Together, these results suggest that *K. brasiliensis* is a natural source of antioxidant and antibacterial agents that can be applied in the research and development of new antibiotics for the treatment of *Salmonella* gastroenteritis because they are able to interfere in the *Salmonella* growth, probably due to cell membrane damage.

## 1. Introduction

Oxidative stress is a well-known event in the cells and tissues related to the action of overproduction of free radicals and reactive metabolites named reactive oxygen species (ROS) and reactive nitrogen species (RNS) and can lead to the damage of cellular molecules such as proteins, lipids, and deoxyribonucleic acid (DNA) [1]. Furthermore, it has been widely documented that increased ROS levels modify cell signaling of host proteins, leading to pathological disorders such as bacterial infections and inflammatory processes [2].

A review of 126 articles focusing on the role of nutrients and phytochemicals in the modulation of antimicrobial resistance (AMR), a serious and alarming public health problem worldwide of the 21^st^ century [3], showed that 40 of them involved antioxidants such as n-acetylcysteine, ambroxol, ascorbic acid, glutathione, and vitamin E [4]. Goswami et al. [5] described that commonly used cellular and dietary antioxidants affect the activity of therapeutic antibiotics. These authors demonstrated that the presence of glutathione increased antibacterial activity of *β*-lactams, revealing that this substance could act as a differential antibiotic susceptibility modulator for bacteria. Moreover, another review reported the use of antioxidants in urinary tract infection and showed that these agents can be effectively used combined with antibiotics to reduce the postpyelonephritic scar formation in correlation to their ability to reduce oxidative stress [6].

In addition, the scenario of AMR and the perspective of new therapeutic approaches to treat multidrug-resistant (MDR) bacterial infections have globally attracted the attention of researchers to the secondary plant metabolites, which may represent an alternative and economically feasible strategy to research, discover, and develop new antibiotics [7]. Among them, the flavonoids, the largest and most important group of phenolic compounds in nature, are considered an essential component in the diversity of medicinal, nutraceutical, pharmaceutical, and cosmetic applications [8]. These compounds are widely found in fruits, vegetables, grains, barks, roots, stems, flowers, and certain beverages like tea and wine [8].

Among the diversity of plant options with expressive flavonoid content, *Kalanchoe* Adans. (Crassulaceae DC.) comprised more than one hundred species and it is widely distributed in tropical areas such as Africa and Brazil [9]. In these places, the fresh leaves juice is traditionally used to treat inflammatory and infectious processes such as dysentery, cholera, gastric ulcers, urinary diseases, and ear and pulmonary infections [9]. *Kalanchoe brasiliensis* Cambess., a native Brazilian plant distributed from Bahia to São Paulo states and often found along the southeast coast, is popularly known as “saião” and traditionally used for its pharmacological properties [10]. The scientific studies reported in the literature described the immunomodulatory [11, 12] and anti-inflammatory activities [10, 12–14], the acetylcholinesterase inhibitory action [15, 16], the antimicrobial effect [17], and the gastroprotective property [18, 19].

Regarding the chemical composition, there were seven glycosylated patuletin-derived flavonoids previously identified from the stems and leaves of this plant species [11], with kalambrosides A, B, and C being firstly described, and patuletin 3-*O*-*α*-L-rhamnopyranosyl-7-*O*-*α*-L-rhamnopyranoside posteriorly reported as a chemical marker of the hydroethanolic extract from *K. brasiliensis* leaves [20]. Subsequently, flavonoid glycosides derived from eupafolin were additionally detected as well as quercetin-*O*-hexoside-*O*-deoxyhexoside [21]. Recently, Araújo et al. [18], using ultra-high-performance liquid chromatography coupled to a mass spectrometer (UHPLC-MS), corroborated the presence of flavonoid glycosides derived from patuletin and eupafolin. The kalanchosine dimalate (kalanchosine (1), 3,6-diamino-4,5-dihydroxyoctanedioic acid, plus malic acid (2)), an anti-inflammatory salt from this plant species, was also reported [10].

Despite the chemical and pharmacological publications presented above, few studies focusing on the possible antioxidant [18] and antimicrobial activities [17] were scientifically cited. Furthermore, the traditional use of *K. brasiliensis* to threat dysentery is noteworthy [9] and the fact that the most severe infectious diarrheas are caused predominantly by bacteria, and among them, nontyphoidal salmonellosis cases are globally widespread [22]. Additionally, according to the global priority pathogens list (global PPL) published by the World Health Organization (WHO) to guide research and development of new antibiotics, the fluoroquinolone-resistant *Salmonella* strains is categorized as a high-priority pathogen, which poses a serious threat to human health [3].

With this context in mind, the present study was aimed at investigating the chemical composition, the *in vitro* antioxidant and antibacterial activities, and the mode of action against *Salmonella* species of the hydroethanolic extracts 30%, 50%, and 70% from *K. brasiliensis* fresh leaves collected in the Brazilian summer and spring seasons looking for new options to treat *Salmonella* gastroenteritis.

## 2. Materials and Methods

### 2.1. Chemicals and Reagents

All chemicals and reagents used (and their sources), including solvents, were of analytical or HPLC grade as follows: acetic acid and pyridine (Vetec Química Farm Ltda, Rio de Janeiro, RJ, Brazil); aluminum chloride (F. Maia, Belo Horizonte, MG, Brazil); chloroform (Labsynth, Diadema, SP, Brazil); ethanol P.A. (99.5%) (Biotec Reagentes Analíticos, Pinhais, PR, Brazil); ampicillin 96.0-100.5% (anhydrous basis), chloramphenicol ≥ 98% (TLC), 2,2-diphenyl-1-picryl-hydrazyl (DPPH), gallic acid, levofloxacin ≥ 98% (HPLC), quercetin, rutin, tannic acid, and Tween 40 (Sigma-Aldrich Chemical Co., St. Louis, MI, USA); sodium carbonate (InLab Diadema, SP, Brazil); Müeller-Hinton agar and Müeller-Hinton Broth (Difco Laboratories®, Detroit, MI, USA); and McFarland scale 0.5 (DME Diagnóstico Microbiológicos Especializados®, São Paulo, SP, Brazil). Purified water was obtained using the Milli-Q Plus® system (Millipore, Milford, MA, USA).

### 2.2. Plant Material

Fresh leaves of *K. brasiliensis* were collected from the medicinal garden of the Faculty of Pharmacy, Universidade Federal de Juiz de Fora, Juiz de Fora city, Minas Gerais state, southeast region of Brazil (21°26′40^″^ S, 43°22′1^″^ W) on January 7 (1^st^ collection; Brazilian Summer) and September 22 (2^nd^ collection; Brazilian Spring) 2016. The plant species did not show blooming when collected. The botanical identification was performed by Dr. Marcus Alberto Nadruz Coelho, researcher at the Instituto de Pesquisa Jardim Botânico do Rio de Janeiro, Rio de Janeiro city, Rio de Janeiro state, Brazil, and a voucher specimen (CESJ no. 43980) was deposited at the Herbarium Leopoldo Krieger, Universidade Federal de Juiz de Fora. The plant name *Kalanchoe brasiliensis* Cambess. has been checked with http://www.theplantlist.org, being a synonym of *Kalanchoe laciniata* (L.) DC. (http://www.theplantlist.org/tpl1.1/record/tro-8902741) (accessed 07 May 2019).

### 2.3. Preparation of Extracts

The hydroethanolic extracts 30%, 50%, and 70% [23] of *K. brasiliensis* fresh leaves collected in January (HEJ30, HEJ50, and HEJ70, respectively) and September (HES30, HES50, and HES70, respectively) 2016 were obtained following the method used by Costa et al. [20]. Fresh leaves (300 g) were extracted with ethanol P.A. (99.5%) at a ratio of 30 : 70 (HEJ30, HES30), 50 : 50 (HEJ50, HES50), and 70 : 30 (HEJ70, HES70) ethanol : water (*v*/*v*) in a plant : solvent proportion of 1 : 1 (*w*/*v*) using a mechanical blender. Then, these hydroethanolic extracts were filtered on a standard filter paper and concentrated by rotary evaporator (R-215 Büchi Labortechnik AG, Flawil, Switzerland) at 45°C.

### 2.4. Chemical Characterization by High-Performance Liquid Chromatography with Diode-Array Detection (HPLC-DAD)

HEJ30, HEJ50, HEJ70, HES30, HES50, and HES70 were evaluated by HPLC-DAD in a chromatograph Agilent Technologies 1200 series. The analysis was performed in reversed phase using Zorbax SB-C18 column (4.6 mm × 150 mm, 5 *μ*m). The mobile phase was a linear gradient from 0 to 30 min with distilled water and methanol HPLC grade (95 : 5 to 10 : 90). The injection volume was 20 *μ*L at the concentration of 1 mg/mL of the extract dissolved in distilled water, and the flow rate remained constant at 0.8 mL/min. Detection was performed by UV-DAD detector set at a wavelength of 254 nm while the UV spectra was acquired by scanning over a range of 190 to 400 nm.

### 2.5. Total Phenolic and Flavonoid Determinations

The total phenolic content was spectrophotometrically determined according to the Folin and Denis [24] protocol using tannic acid as the standard. HEJ30, HEJ50, HEJ70, HES30, HES50, and HES70 were dissolved in distilled water (2 mg/mL), and an aliquot of 300 *μ*L was added to the Folin-Denis reagent (500 *μ*L) and distilled water (500 *μ*L). After 30 min, saturated sodium carbonate (500 *μ*L) was added to neutralize the reaction. The absorbance was recorded at 760 nm in a microplate reader (Thermo Scientific™, Multiskan™ Sky Microplate Spectrophotometer, Waltham, MA, USA) using distilled water as a blank. The analysis was carried out in triplicate, and the results were expressed as milligram of tannic acid equivalent per gram of extract (mgTAE/g).

The spectrophotometric method was applied for total flavonoid determination based on the formation of acid stable complexes with aluminum chloride using rutin as the standard [25]. For this quantification, HEJ30, HEJ50, HEJ70, HES30, HES50, and HES70 were dissolved in a mixture of ethanol : water (3 : 7) at different concentrations, added into a tube containing ethanol, acetic acid, pyridine (20% in ethanol, *v*/*v*), and aluminum chloride hexahydrated (8% in ethanol, *w*/*v*), and the volume was completed to 5000 *μ*L with distilled water. After 30 min, the absorbance was measured at 412 nm using a spectrophotometer (Shimadzu®, UV-1800, Tokyo, Japan). The analysis was performed in triplicate, and the results were expressed as milligram of rutin equivalent per gram of extract (mgRE/g).

### 2.6. Antioxidant Activity

#### 2.6.1. DPPH Radical Scavenging Activity

The free radical scavenging activity of HEJ30, HEJ50, HEJ70, HES30, HES50, and HES70 was determined based on their ability to react with a stable DPPH free radical following the method described by Blois [26]. The hydroethanolic extract solutions (0.49 to 500 *μ*g/mL) were prepared and mixed with an equal volume of methanol solution of DPPH (0.03 mM). After 60 min at room temperature and protected from light, the absorbance was measured in a microplate reader (Thermo Scientific™, Multiskan™ Sky Microplate Spectrophotometer, Waltham, MA, USA) at 517 nm. The experiment was performed in triplicate. Ascorbic acid and quercetin were used as standards. The antioxidant capacity of HEJ30, HEJ50, HEJ70, HES30, HES50, and HES70 was expressed as the 50% effective concentration (EC_50_), which was defined as the concentration (*μ*g/mL) of extract required to reduce 50% of DPPH or to obtain 50% antioxidant effect [27].

#### 2.6.2. Phosphomolybdenum Reducing Power Assay

The total antioxidant capacity of HEJ30, HEJ50, HEJ70, HES30, HES50, and HES70 was also evaluated by phosphomolybdenum reducing power assay according to Prieto et al. [28], using ascorbic acid as the standard. This spectrophotometric method is based on the reduction of molybdenum (VI) to molybdenum (V) in the presence of certain substances with antioxidant capacity, with formation of phosphate/molybdenum (V) green complex at acidic pH [28]. The hydroethanolic extract solutions (0.2 mg/mL) were added to 2000 *μ*L of the phosphomolybdenic complex reagent and kept in a water bath at 95°C for 90 min. After cooling, 250 *μ*L was transferred into a 96-well microplate, and absorbance was measured in a microplate reader (Thermo Scientific™, Multiskan™ Sky Microplate Spectrophotometer, Waltham, MA, USA) at 695 nm. The experiment was performed in triplicate. The results were expressed as the relative antioxidant activity of ascorbic acid (%RAA ascorbic acid) ± standard deviation (SD).

#### 2.6.3. *β*-Carotene Bleaching Assay

The antioxidant activity of HEJ30, HEJ50, HEJ70, HES30, HES50, and HES70 was additionally investigated by *β*-carotene/linoleic acid system method as described by Melo and Mancini-Filho [29], with little adjustments. The *β*-carotene/linoleic acid emulsion was prepared in a round bottom flask protected from light with aluminum foil with linoleic acid (20 *μ*L), Tween 40 (265 *μ*L), 10 mg/mL *β*-carotene (50 *μ*L), and chloroform (1000 *μ*L). After mixing, the chloroform was evaporated using a rotary evaporator (TECNAL, Piracicaba, SP, Brazil) at 40°C, and distilled water previously saturated with oxygen for 30 min was added (20,000 *μ*L). The emulsion had the absorbance adjusted to 0.6-0.7 at 470 nm. Then, 38.4 *μ*g/mL HEJ30, HEJ50, HEJ70, HES30, HES50, and HES70 and quercetin (10 *μ*L) were placed into a 96-well microplate containing 250 *μ*L of the emulsion and maintained at 45°C for 120 min. The reaction was monitored by discoloration of *β*-carotene by absorbance reduction measurement at 470 nm, with reading at 15 min intervals for a total of 120 min, using a microplate reader (Thermo Scientific™, Multiskan™ Sky Microplate Spectrophotometer, Waltham, MA, USA), in triplicate. The percentage of inhibition (%I) of lipid peroxidation was calculated.

### 2.7. In Vitro Antibacterial Activity

#### 2.7.1. Bacterial Strains

*Staphylococcus aureus* subsp. *aureus* (methicillin-sensitive *Staphylococcus aureus* (MSSA)) (ATCC® 6538™ and ATCC® 29213™), *Escherichia coli* (ATCC® 10536™ and ATCC® 25922™), *Salmonella enterica* subsp. *enterica* serovar Choleraesuis (ATCC® 10708™), *Salmonella enterica* subsp. *enterica* serovar Typhimurium (ATCC® 13311™), and *Pseudomonas aeruginosa* (ATCC® 9027™ and ATCC® 27853™) reference strains and methicillin-resistant *Staphylococcus aureus* (MRSA) 1485279, 1605677, 1664534, 1688441, and 1830466; *Salmonella* Enteritidis 1406591, 1418594, and 1628260; and *Salmonella* spp. 1266695 and 1507708 (fluoroquinolone-resistant) clinical strains were selected for the *in vitro* antibacterial activity assessment. The American Type Culture Collection (ATCC®) reference strains were obtained from the Instituto Nacional de Controle de Qualidade em Saúde (INCQS), Fundação Oswaldo Cruz (Fiocruz), Rio de Janeiro, Brazil. The clinical strains were isolated from the blood (MRSA) and the blood or urine (*Salmonella* spp. and *Salmonella* Enteritidis, respectively) of patients who attended at Hospital Universitário Clementino Fraga Filho, Universidade Federal do Rio de Janeiro, Rio de Janeiro city, Rio de Janeiro state, Brazil. These strains were identified by a VITEK® 2 automated system (bioMérieux, Durham, NC, USA). The ATCC® and clinical strains were stored as suspensions in a 10% (*w*/*v*) skim milk solution containing 10% (*v*/*v*) glycerol at -20°C. Prior to use in the bioassays, these strains were aerobically grown in Müeller-Hinton agar (MHA) at 35 ± 2°C for 18-24 h. In this article, we used *S. aureus* (ATCC® 6538), *S. aureus* (ATCC® 29213), *E. coli* (ATCC® 10536), *E. coli* (ATCC® 25922), *S.* Choleraesuis (ATCC® 10708), *S.* Typhimurium (ATCC® 13311), *P. aeruginosa* (ATCC® 9027), *P. aeruginosa* (ATCC® 27853), MRSA 1485279, MRSA 1605677, MRSA 1664534, MRSA 1688441, MRSA 1830466, *S.* Enteritidis 1406591, *S.* Enteritidis 1418594, *S.* Enteritidis 1628260, *Salmonella* spp. 1266695, and *Salmonella* spp. 1507708 to simplify the text.

#### 2.7.2. Determination of Minimal Inhibitory Concentration (MIC) and Minimal Bactericidal Concentration (MBC)

The broth microdilution method following the Clinical and Laboratory Standards Institute (CLSI) guideline M07-A9 [30] with little adjustments was employed to determine MIC values of HEJ30, HEJ50, HEJ70, HES30, HES50, HES70, ampicillin (AMP), chloramphenicol (CHL), and, when applicable, levofloxacin (LEV), against ATCC® and clinical strains described above. The hydroethanolic extract (12.5 mg/mL (*w*/*v*)) and antibiotic stock solutions (1.25 mg/mL (*w*/*v*)) were prepared in sterile distilled water and in solvents and diluents recommended by the M100-S24 document [31], respectively. In a sterile 96-well microplate, twofold serial dilutions of hydroethanolic extracts (quadruplicate) and antibiotics (triplicate) were prepared in Müeller-Hinton broth (MHB) at concentrations ranging from 40 to 5000 *μ*g/mL and 4 to 500 *μ*g/mL, in this order. MIC values above 5000 *μ*g/mL were not determined. Subsequently, 10 *μ*L of standardized bacteria suspension according to the 0.5 McFarland scale were added. After incubation at 35 ± 2°C for 16 to 20 h under aerobic conditions, 20 *μ*L of 1 mg/mL 2,3,5-triphenyl tetrazolium chloride (TTC) solution (*w*/*v*) was used as an indicator of bacterial growth (any color change from purple to pink was recorded as bacterial growth). Then, the system was incubated for further 30 min, and the MIC was determined. The appropriate controls were performed. After determination of MIC values, MBC was established according to Andrews' method [32] by spreading of 10 *μ*L of suspensions from wells showing no visible bacterial growth on MHA Petri dishes. After incubation at 35 ± 2°C for 16 to 20 h under aerobic conditions, the presence or absence of bacterial growth was analyzed. MBC was determined as the lowest concentration of dilutions that prevented the visible bacterial growth after subculture on MHA Petri dishes. Bacterial growth or no bacterial growth on MHA revealed a bacteriostatic or bactericidal effect, respectively.

#### 2.7.3. Time-Kill Assay

The time-kill curves of HEJ70 and HES70 (0.5 × *MIC*, 1 × *MIC*, and 1.5 × *MIC*) and levofloxacin (LEV) (1 × MIC) were carried out for *S.* Choleraesuis (ATCC® 10708), *S.* Typhimurium (ATCC® 13311), and *Salmonella* spp. 1507708 as recommended by da Silva et al. [33]. MHB containing 5 × 10^5^ CFU/mL of bacterial cultures and HEJ70 or HES70 or LEV (positive control) were aerobically incubated at 35 ± 2°C. Growth control (GC = MHB + bacterial inoculum) was also prepared and incubated at the same conditions. Optical density (OD) was spectrophotometrically recorded at 640 nm before incubation (*t* = 0) and every 60 min for 12 h, and the final measurement was performed at 24 h. The bacterial growth curve was constructed as a function of the OD_640_ variation over time. The experiments were performed in triplicate.

### 2.8. Mode of Action of Hydroethanolic Extracts from *Kalanchoe brasiliensis* Fresh Leaves against *Salmonella* Species

#### 2.8.1. Bacterial Cell Viability Assay

Bacterial cell viability in the presence of hydroethanolic extracts and LEV was determined according to da Silva et al. [33]. One mL of the subcultured bacterial cells (OD_610_ = 0.7) of *S.* Choleraesuis (ATCC® 10708), *S.* Typhimurium (ATCC® 13311), and *Salmonella* spp. 1507708 resuspended in 0.9% sterile saline solution was added to 19 mL of sterile phosphate buffer pH = 7.1 (50 mM) with HEJ70 or HES70 (0.5 × MIC, 1 × MIC, and 1.5 × MIC) or LEV (1 × MIC). After 1 h at 35 ± 2°C, an aliquot in the order of dilution of 10^5^ was inoculated into Petri dishes containing MHA and incubated at 35 ± 2°C, aerobically, for 18 to 20 h. The results were graphically expressed as CFU/mL × 10^5^.

#### 2.8.2. Leakage of Compounds Absorbing at 280 nm

Bacterial cell suspensions of *S.* Choleraesuis (ATCC® 10708), *S.* Typhimurium (ATCC® 13311), and *Salmonella* spp. 1507708 were prepared as described above (2.8.1) and used to spectrophotometrically measure the extravasation of compounds absorbing at 280 nm (loss of proteins and intracellular genetic material) in the supernatant as reported by Kim et al. [34]. One mL of the bacterial suspensions treated with HEJ70 or HES70 (0.5 × MIC, 1 × MIC, and 1.5 × MIC) or LEV (1 × MIC) was aerobically incubated for 1 h at 35 ± 2°C. Cell supernatants were obtained by centrifugation (10,000 × g per 10 min) and the absorbance was spectrophotometrically determined at 280 nm. The release of compounds absorbing at 280 nm was graphically expressed as a relative ratio of OD_280_ of HEJ70 or HES70 or LEV treated and untreated cells.

### 2.9. Statistical Analysis

Data were expressed as mean ± S.D. or S.E.M. Statistical significance was determined by one-way ANOVA analysis of variance followed by the Tukey test. *p* < 0.05 was considered significant.

## 3. Results and Discussion

The results of weight, yield, and total phenolic and flavonoid contents obtained with HEJ30, HEJ50, HEJ70, HES30, HES50, and HES70 are shown in Table 1. According to this Table, discrete variations in mass and yield values were noted, being the largest results observed when ethanol 50% and 70% were used. Similar results were reported by Seo et al. [23] who investigated the effects of water, ethanol, methanol, and different concentrations of hydroethanolic solvents on total phenolic and flavonoid contents and antioxidant activity of extracts from guava (*Psidium guajava* L.) leaves to establish the best extraction solvent for use with this plant. These authors described that the total phenolic content of hydroethanolic extract 50% of guava leaves was higher than 30%, 70%, and 90%, while the total flavonoid content of hydroethanolic extract 70% was higher than 30%, 50%, and 90%. As displayed in Table 1, the highest values of total phenolic and flavonoid contents were obtained with HEJ70 (30.11 ± 0.27 mgTAE/g and 16.95 ± 0.05 mgRE/g, respectively) and HES70 (27.06 ± 0.10 mgTAE/g and 13.33 ± 0.05 mgRE/g, in this order). Our data were supported by Muzitano et al. [35], who standardized the extract of *Kalanchoe pinnata* leaves, and reported that active flavonoids were significantly more abundant when the leaves were collected in the summer. As can be observed in Table 1, the total flavonoid content of hydroethanolic extracts of *K. brasiliensis* leaves collected in the summer (HEJ30, HEJ50, and HEJ70) increased when compared with those obtained in the spring (HES30, HES50, and HES70).

**Figure 1:**
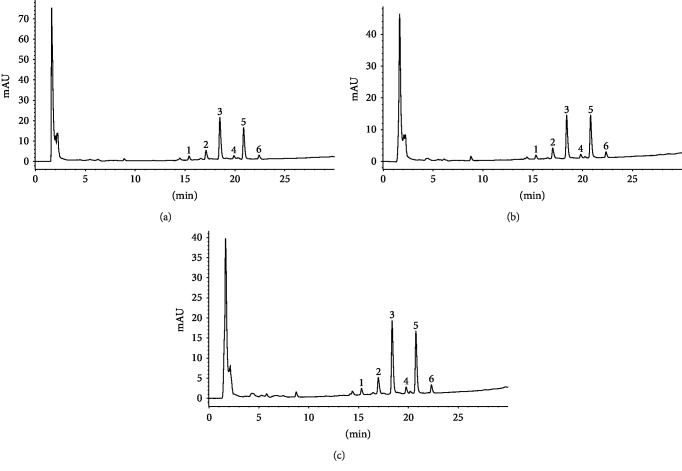
Chromatographic profiles of the hydroethanolic extract (a) 30% (HEJ30), (b) 50% (HEJ50), and (c) 70% (HEJ70) from *Kalanchoe brasiliensis* fresh leaves collected in January obtained by HPLC-DAD at 254 nm showing the presence of patuletin and eupafolin derivatives. Peak 1: flavonol indicating a methylated or glycosylated patuletin; peak 2: flavonol indicating a methylated or glycosylated patuletin; peak 3: flavone suggesting eupafolin or derivative or flavonol indicating a methylated or glycosylated patuletin; peak 4: flavone suggesting eupafolin or derivative; peak 5: flavonol indicating a methylated or glycosylated patuletin; and peak 6: flavone suggesting eupafolin or derivative.

**Figure 2:**
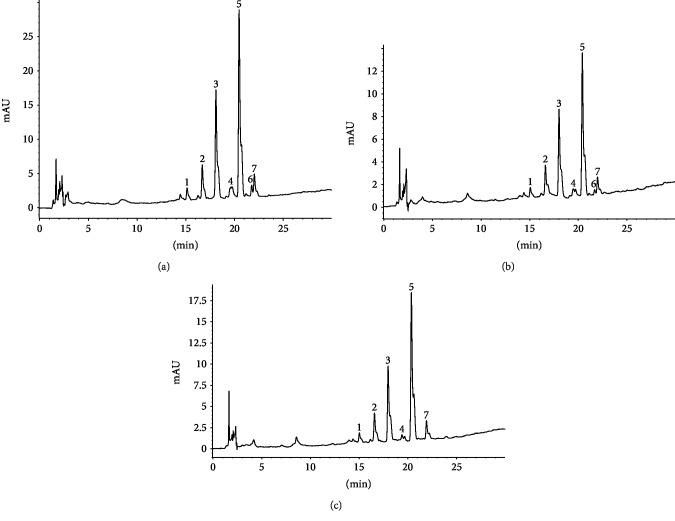
Chromatographic profiles of the hydroethanolic extract (a) 30% (HES30), (b) 50% (HES50), and (c) 70% (HES70) from *Kalanchoe brasiliensis* fresh leaves collected in September obtained by HPLC-DAD at 254 nm showing the presence of patuletin and eupafolin derivatives. Peak 1: flavonol indicating a methylated or glycosylated patuletin; peak 2: flavonol indicating a methylated or glycosylated patuletin; peak 3: flavonol indicating a methylated or glycosylated patuletin; peak 4: flavonol indicating a methylated or glycosylated patuletin; peak 5: flavonol indicating a methylated or glycosylated patuletin; peak 6: flavone suggesting eupafolin or derivative or flavonol indicating a methylated or glycosylated patuletin; and peak 7: flavone suggesting eupafolin or derivative.

For better understanding of the phytochemicals of *K. brasiliensis*, the chemical profiles of HEJ30, HEJ50, HEJ70, HES30, HES50, and HES70 were established by HPLC-DAD, where the chromatograms at 254 nm exhibited six main peaks (1, 2, 3, 4, 5, and 6 or 7), as depicted in Figure 1 (HEJ30, HEJ50, and HEJ70) and Figure 2 (HES30, HES50, and HES70). The data observed by UV-Vis spectrum analysis of these extracts suggested the presence of flavonoids, characterized by two absorbance bands A and B [36]. According to Costa et al. [20], flavonoids could be considered chemical markers of *K. brasiliensis*, and as aforementioned, the majority of these compounds are correlated to glycosylated patuletin derived flavonoids and also derivatives of the flavonoid aglycone eupafolin, with expressive presence of *O*-glycosylated flavonoids [11, 18, 20, 21]. It is noteworthy to reinforce that patuletin 3-*O*-*α*-L-rhamnopyranosyl-7-*O*-*α*-L-rhamnopyranoside, the main chemical marker of *K. brasiliensis*, like other patuletin derivates, are not commercially available [20]. These compounds belong to the subclasses of flavones and flavonols (3-hydroxyflavones (flavonols) or flavonols with substituted 3-hydroxyl groups (methylated or glycosylated)). Additionally, Tsimogiannis et al. [36] wrote that band A is observed at 310-350 nm for flavones, while for flavonols, it is between 350 and 385 nm. Band B found in the 250-290 nm range is much the same in these flavonoid subclasses (Supplementary Materials Figures [Supplementary-material supplementary-material-1] and [Supplementary-material supplementary-material-1]).

The *in vitro* antioxidant activity of HEJ30, HEJ50, HEJ70, HES30, HES50, and HES70 was evaluated by DPPH radical scavenging, phosphomolybdenum reducing power, and *β*-carotene bleaching assays. As can be seen in Table 2, in the DPPH assay, among the six hydroethanolic extracts investigated, HEJ50 (EC_50_54.66 ± 2.64 *μ*g/mL) showed the best scavenging activity, followed by HEJ70 (EC_50_88.68 ± 2.79 *μ*g/mL), HEJ30 (EC_50_99.78 ± 1.67 *μ*g/mL), HES50 (EC_50_102.00 ± 2.23 *μ*g/mL), HES70 (EC_50_113.60 ± 4.34 *μ*g/mL), and HES30 (EC_50_248.40 ± 1.31 *μ*g/mL). Seo et al. [23], also using this method among others to investigate the antioxidant ability of hydroethanolic extracts of guava leaves, demonstrated that the activity of 50% hydroethanolic extract was more expressive than 30%, 70%, and 90%. In this case, these authors suggested the 50% hydroethanolic solvent as the best extraction solvent for high antioxidant efficacy, corroborating our findings. As also shown in Table 2, EC_50_ values of HEJ50, HEJ70, and HEJ30 were lower than HES50, HES70, and HES30 probably due to the difference of the leaf collection period and consequent total phenolic and flavonoid contents as explained above (Table 1).

The results obtained by phosphomolybdenum reducing power demonstrated that the hydroethanolic extracts presented a weak capacity when compared with ascorbic acid (Table 2). HEJ70 (16.78 ± 0.24% RAA ascorbic acid) and HEJ50 (16.73 ± 0.34% RAA ascorbic acid) were more effective to reduce Mo (VI) to Mo (V) while the lowest effect was shown by HES30 (8.49 ± 0.23% RAA ascorbic acid). Thus, in this method, the *in vitro* antioxidant activity was found in the following order: HEJ70 = HEJ50 > HES70 > HES50 > HEJ30 > HES30, supporting the influence of the percentage of solvent in the efficiency of the antioxidant capability. This data confirmed the DPPH results and showed the ability of the phenolic compounds and flavonoids to reduce both free radicals [37].

The lipid peroxidation method is related to the generation of free radicals in human diseases which play a significant pathological role. For example, peroxidation appears to be important in atherosclerosis and in worsening the initial tissue injury caused by ischemic or traumatic brain damage and neurodegenerative disorders [38]. Additionally, cytoplasmic membrane lipid peroxidation, an autooxidative process caused by the attack of free radicals, contributes to the progression of various types of regulated cell death [39]. Considering these biological data, our results displayed that HEJ70 and HES70 were more active, since they inhibited the oxidation of linoleic acid and subsequent bleaching of *β*-carotene, with %I values equal to 44.46 ± 0.84 and 41.85 ± 0.39, respectively (Table 2). So, these hydroethanolic extracts possess compounds able to inhibit the lipid peroxidation, and this capacity can be relevant in the prevention of the oxidative damage.

Some pathologies arising during infection can be attributed to oxidative stress, and generation of ROS in the infectious process can even have fatal consequences [40]. In this sense, antioxidants have the roles to neutralize the excess of free radicals, to protect the cells against their toxic effects, and to contribute to disease prevention [41]. The combination of the antioxidant and antibacterial activities of medicinal plants can be better exploited to manage various disorders, mainly infectious processes. In this way, the results of the *in vitro* antibacterial activity of HEJ30, HEJ50, HEJ70, HES30, HES50, and HES70 by MIC are displayed in Table 3. This Table shows that, among these extracts, HEJ70 and HES70 were the most active, since they were able to inhibit *S. aureus* (ATCC® 6538); *E. coli* (ATCC® 25922); *S.* Choleraesuis (ATCC® 10708); *S.* Typhimurium (ATCC® 13311); MRSA 1605677 and 1830466; *S.* Enteritidis 1406591, 1418594, and 1628260; and *Salmonella* spp. 1266695 and 1507708, with MIC values of 5000 *μ*g/mL and bacteriostatic effect. MBC was not determined because 5000 *μ*g/mL was the highest concentration evaluated. The antibiotic effect was classified based on Kuete's criteria where the antimicrobial activity of plant extracts was categorized as significant (MIC < 100 *μ*g/mL), moderate (100 < MIC ≤ 625 *μ*g/mL), or weak (MIC > 625 *μ*g/mL) [42]. However, in an ethnopharmacological survey, Fabry et al. [43] concluded that plants which are used in traditional medicine against infections may have some antimicrobial activity with MICs < 8000 *μ*g/mL. So, *K. brasiliensis* possesses a weak antibacterial activity but good enough to explain its use in traditional medicine to treat infectious processes [9] as previously mentioned.

Taking into account that this medicinal plant is widely use in northeastern Brazil for the treatment of wounds, boils, and abscesses [44], and the fact that *S. aureus* is the leading cause of bacterial infections involving skin and soft tissue among other disorders [45], *S. aureus* (ATCC® 6538), *S. aureus* (ATCC® 29213), and five MRSA clinical strains were evaluated as shown in Table 3. These MRSA strains were previously investigated by da Costa et al. [46] focusing on the analysis of the clinical and microbiological characteristics of heteroresistant (hVISA) and vancomycin-intermediate *S. aureus* (VISA) from bloodstream infections (BSI) in a Brazilian teaching hospital. These authors demonstrated the multidrug-resistant (MDR) profile of these strains and corroborated the relevance of the antibiotic effect of the hydroethanolic extracts tested. This antibacterial activity was previously described by Silva et al. [17] that reported the bacteriostatic effect of 4% and 8% essential oils of *K. brasiliensis* leaves against *S. aureus* (ATCC® 6538), *S. aureus* (ATCC® 25923), and ten *S. aureus* clinical strains, including MRSA. With regard to the mechanism of action, Babii et al. [47] evaluated the effect of the synthetic tricyclic flavonoid 1 against *S. aureus* (ATCC® 25923) and described that the mode of action is related to the impairment of the cell membrane integrity and to cell agglutination. It is noteworthy that *S. aureus* (ATCC® 25923) is a MSSA and a biofilm forming strain. Furthermore, ultrastructural changes showed that the flavonoid artonin I was able to induce destruction through cell membrane damage and abnormal division pattern alteration on MDR-*S. aureus* strains [48]. Probably, the flavonoids detected in HEJ30, HEJ50, HEJ70, HES30, HES50, and HES70 possess a similar mode of action against *S. aureus* (ATCC® 6538), *S. aureus* (ATCC® 29213), and the five MRSA clinical strains tested.

*K. brasiliensis* is also traditionally used to treat dysentery, an acute diarrheal infection that promotes a loss of water and electrolytes with a clinical scenario of abdominal cramping, fever, and bloody and/or mucoid stools, mainly caused by the *Salmonella* genus [9, 22, 49]. As depicted by Table 3, HEJ30, HEJ50, HEJ70, HES30, HES50, and HES70 were active against all ATCC® and clinical *Salmonella* strains tested. However, Silva et al. [17] described that the hydroethanolic extracts and essential oil of *K. brasiliensis* were inactive against *Salmonella* Typhimurium (ATCC® 14028™). So, to clarify this antibacterial activity and evaluate the mode of action, time-kill, bacterial cell viability, and leakage of compounds absorbing at 280 nm assays were carried out using HEJ70 and HES70 (0.5 × *MIC*, 1 × *MIC*, and 1.5 × *MIC*), since these extracts were the most active, including against *S.* Choleraesuis (ATCC® 10708), *S.* Typhimurium (ATCC® 13311), and *Salmonella* spp. 1507708 (a fluoroquinolone-resistant *Salmonella* spp. clinical isolate).

**Figure 3:**
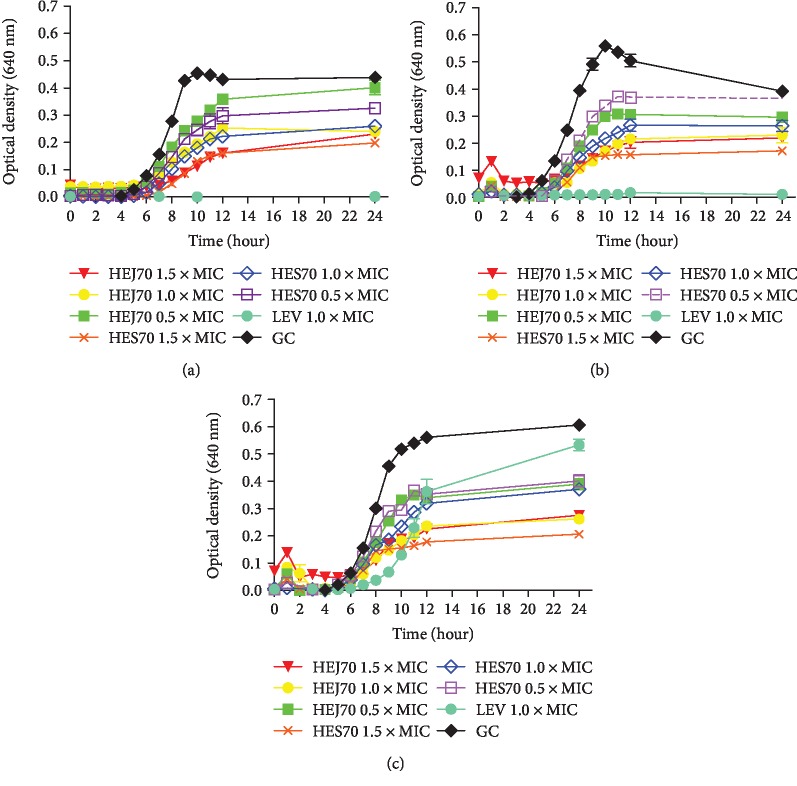
Time-kill curves of the hydroethanolic extract 70% from *Kalanchoe brasiliensis* fresh leaves collected in January (HEJ70) and September (HES70) and levofloxacin (LEV) against (a) *Salmonella* Choleraesuis (ATCC® 10708), (b) *Salmonella* Typhimurium (ATCC® 13311), and (c) *Salmonella* spp. 1507708 during 24 h of incubation. Experiments were performed in triplicate.

According to Figure 3, HEJ70 and HES70 clearly reduced the bacterial multiplication rate in a concentration-dependent manner when compared with the GC, extending the *lag* phase from 4 to 5 h (*S.* Typhimurium (ATCC® 13311)) and from 4 to 6 h (*S.* Choleraesuis (ATCC® 10708) and *Salmonella* spp. 1507708). The bacteriostatic effect, previously observed by MBC determination, was also preserved during 24 h of incubation for these three *Salmonella* strains. The increased *lag* period of the growth of these three *Salmonella* strains in the presence of HEJ70 and HES70 seems to be a result of the killing of the majority of the bacteria. HEJ70 and HES70 were more active than LEV against *Salmonella* spp. 1507708, demonstrating its resistance as detected by the VITEK® 2 system and the inability to express the bactericidal effect, different from that observed with *S.* Choleraesuis (ATCC® 10708) and *S.* Typhimurium (ATCC® 13311). Figure 3 also shows that HES70 (1.5 × MIC) was the most effective extract against the three *Salmonella* strains. These findings are confirmed by Chimnoi et al. [50], who described the antibacterial activity and the mode of action of essential oil from *Ocimum gratissimum* leaves (OGEO) against *S.* Typhimurium TISTR 292 (ATCC® 13311). In addition, the effect of phenolic compounds on the growth of *S.* Typhimurium (ATCC® 14028) was reported by Pacheco-Ordaz et al. [51], which was related to the concentration and bacterial cell structure. These compounds can bind to bacterial cell membranes, and some of them can interact with lipids and proteins, altering membrane permeability, and they can also interfere with bacterial *quorum sensing* [51]. These findings were previously confirmed by Herrerias et al. [52] who described the cytotoxic effect of eupafolin that can partially be explained by alterations promoted on biological membrane properties. These reports support the possible mode of action of the detected phenolic compounds, mainly flavonoids, in HEJ70 and HES70.

**Figure 4:**
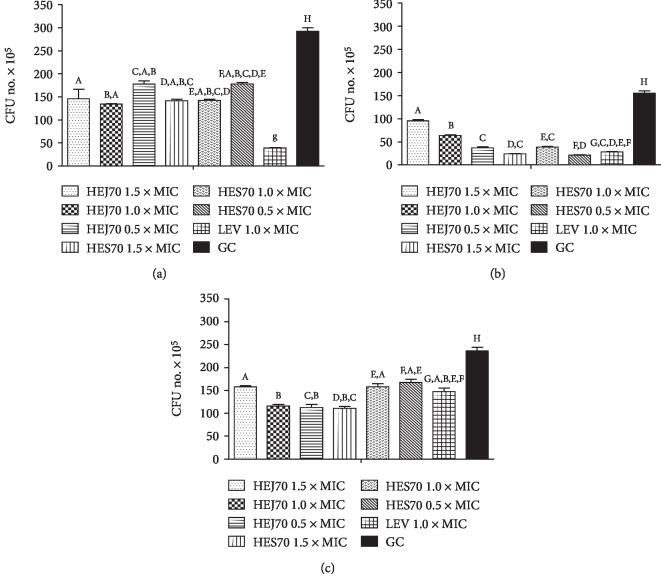
Results of the bacterial cell viability assay after exposure to hydroethanolic extract 70% from *Kalanchoe brasiliensis* fresh leaves collected in January (HEJ70) and September (HES70) and levofloxacin (LEV) against (a) *Salmonella* Choleraesuis (ATCC® 10708), (b) *Salmonella* Typhimurium (ATCC® 13311), and (c) *Salmonella* spp. 1507708. CFU: colony forming unit. Results were expressed in mean ± S.E.M. (*n* = 3). Equal letters in the bars mean no difference (*p* < 0.05) after variance analysis followed by Tukey's test.

The mode of action of HEJ70 and HES70 was initially investigated through the bacterial cell viability assay by the colony forming unit number per mL (CFU/mL) measurement (the CFU number able to survive exposure to the extracts and antibiotic after 1 hour (*lag* phase)). Our results revealed that the three *Salmonella* strains suffered the effect of these extracts which were able to reduce the CFU/mL, but *Salmonella* spp. 1507708 was more sensitive than *S.* Choleraesuis (ATCC® 10708) and *S.* Typhimurium (ATCC® 13311), because HEJ70 and HES70 were more effective when compared with LEV (Figure 4). This reduction can occur by bacterial cell membrane and/or membrane permeability alterations, since Chimnoi et al. [50] reported that 90% of the reduction of green light in *S.* Typhimurium (ATCC® 13311) produced severe damage of the membrane, indicating an alteration of the membrane permeability of this strain. Moreover, Mirzoeva et al. [53] previously described that the flavonoids quercetin and naringenin affected bacterial membrane potential, increasing its permeability. Other flavonoids, such as (-)-epigallocatechin gallate and (-)-epicatechin [54], the synthetic 2,4,2′-trihydroxy-5′-methylchalcone [55] and licochalcones C and D (retrochalcones) [56], and sophoraflavanone G [57], among other subclasses of these compounds, have acted over the bacterial membrane and/or reduced the membrane fluidity. Thus, the flavonoids probably belonging to the subclasses of flavones and flavonols detected in HEJ70 and HES70 may act in the same way, being responsible for the antibiotic effect of these extracts.

**Figure 5:**
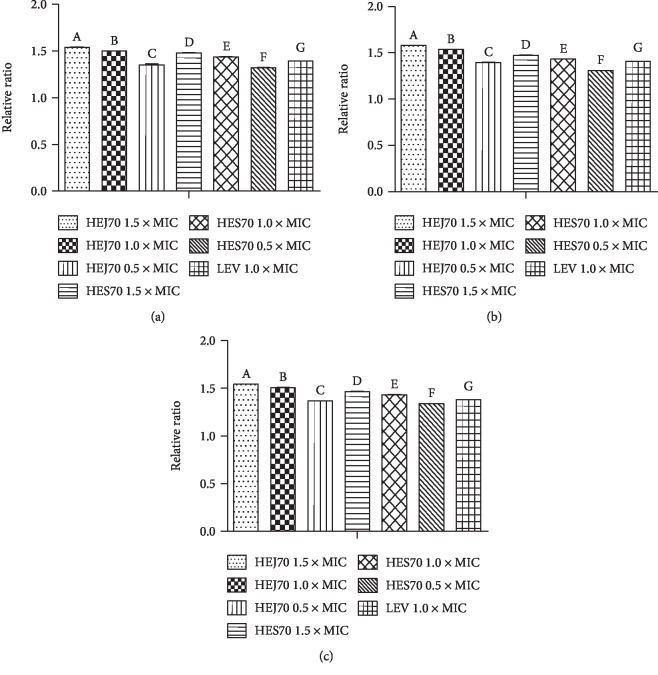
Results of the leakage of compounds absorbing at 280 nm assay expressed as a relative ratio of OD_280_ of the hydroethanolic extract 70% from *Kalanchoe brasiliensis* fresh leaves collected in January (HEJ70) and September (HES70) and treated cells and levofloxacin (LEV) to the one untreated cell of (a) *Salmonella* Choleraesuis (ATCC® 10708), (b) *Salmonella* Typhimurium (ATCC® 13311), and (c) *Salmonella* spp. 1507708. Results were expressed in mean ± S.E.M. (*n* = 3). Equal letters in the bars mean no difference (*p* < 0.05) after variance analysis followed by Tukey's test.

The leakage of bacterial cell contents absorbing at 280 nm which indicates cell lysis and nonselective pore formation, demonstrating the membrane damage [58], was also carried out. According to Kim et al. [34], this leakage is mainly related to the loss of cell proteins that are normally retained by the cell membrane. Actually, as described by Bajpai et al. [59], measurement of specific cell leakage markers, including proteins and 260 nm absorbing materials such as nucleic acid, is an indicator of membrane integrity to a specific antibiotic in relationship to untreated cells. As shown in Figure 5, the results displayed that there was a relevant release of bacterial cell content absorption at 280 nm from *S.* Choleraesuis (ATCC® 10708), *S.* Typhimurium (ATCC® 13311), and *Salmonella* spp. 1507708 when these strains were treated with HEJ70 and HES70 in a concentration-dependent manner. Sadiq et al. [60] demonstrated a rapid loss of proteins and nucleic acids from *S.* Typhimurium human clinical isolates due to irreversible damage to the cytoplasmic membranes in the function of the phenolic content and in the presence of phenolic acids in *Acacia nilotica*. Additionally, Tagousop et al. [61] investigated the mechanism of antibacterial action of flavonoid glycosides from *Graptophyllum grandulosum* and concluded that their mode of antibacterial activity is due to cell lysis and disruption of the cytoplasmic membrane upon membrane permeability. Therefore, the phenolic compounds, mainly flavonoids, detected in HEJ70 and HES70 could be related to the membrane damage.

## 4. Conclusions

*K. brasiliensis*is a natural source of flavonols and flavones and showed promising antioxidant and antibacterial agents. The antibacterial action against *Salmonella* strains is related to the cell membrane integrity and/or permeability alterations. Our data are relevant to research and develop possible novel and innovative antibiotics for therapeutic applications and to propose new approaches to manage infectious processes mainly against *Salmonella* spp.

## Figures and Tables

**Table 1:** Weight, yield, and total phenolic and flavonoid contents of the hydroethanolic extract 30%, 50%, and 70% of *Kalanchoe brasiliensis* fresh leaves collected in January (HEJ30, HEJ50, and HEJ70) and September (HES30, HES50, and HES70).

Month/year	Extract	Weight (g)	Yield (%)	Total phenolic content (mgTAE/g)	Total flavonoid content (mgRE/g)
January/2016	HEJ30	10.32	3.44	26.97 ± 0.27^a^	16.59 ± 0.18^a^
	HEJ50	12.43	4.14	28.16 ± 0.07^b^	15.44 ± 0.19^b^
	HEJ70	12.34	4.11	30.11 ± 0.27^c^	16.95 ± 0.05^c^

September/2016	HES30	9.11	3.04	25.47 ± 0.24^d^	11.84 ± 0.00^d^
	HES50	10.21	3.40	26.17 ± 0.02^e^	12.49 ± 0.10^e^
	HES70	10.81	3.60	27.06 ± 0.11^f,a^	13.33 ± 0.05^f^

The results are expressed as mean ± SD. Total phenolic and flavonoid contents were expressed as tannic acid equivalent (mgTAE/g) and rutin equivalent (mgRE/g), respectively. Equal letters in the same column mean no difference (*p* < 0.05) after variance analysis followed by Tukey's test.

**Table 2:** *In vitro* antioxidant activity of the hydroethanolic extracts 30%, 50%, and 70% from *Kalanchoe brasiliensis* fresh leaves collected in January (HEJ30, HEJ50, and HEJ70) and September (HES30, HES50, and HES70) by 2,2-diphenyl-1-picryl-hydrazyl (DPPH) radical scavenging, phosphomolybdenum reducing power, and *β*-carotene bleaching assays.

Extract/standard substance	DPPH radical scavenging	Phosphomolybdenum reducing power	*β*-Carotene bleaching assay
	EC_50_ (*μ*g/mL)	% RAA ascorbic acid	Inhibition of lipid peroxidation (%)
HEJ30	99.78 ± 1.67^a^	10.08 ± 0.23^a^	40.67 ± 0.50^a^
HEJ50	54.66 ± 2.64^b^	16.73 ± 0.34^b^	39.84 ± 0.59^b^
HEJ70	88.68 ± 2.79^c^	16.78 ± 0.24^c,b^	44.46 ± 0.84^c^
HES30	248.40 ± 1.31^d^	8.49 ± 0.23^d^	41.04 ± 0.42^d^
HES50	102.00 ± 2.23^e,a^	13.04 ± 0.36^e^	37.51 ± 0.47^e^
HES70	113.60 ± 4.34^f^	14.81 ± 0.35^f^	41.85 ± 0.39^f^
Ascorbic acid	0.52 ± 0.04^g^	Not determined	Not determined
Quercetin	0.40 ± 0.10^h^	Not determined	92.33 ± 0.67^g^

Values are expressed as mean ± SD; the experiment was made in triplicate. Equal letters in the same column mean no difference (*p* < 0.05) after variance analysis followed by Tukey's test.

**Table 3:** Minimal inhibitory concentration values of the hydroethanolic extracts 30%, 50%, and 70% from *Kalanchoe brasiliensis* fresh leaves collected in January (HEJ30, HEJ50, and HEJ70) and September (HES30, HES50, and HES70), ampicillin (AMP), chloramphenicol (CHL), and levofloxacin (LEV) against reference and clinical bacterial strains.

Bacterial strain	Minimal inhibitory concentration (MIC) (*μ*g/mL)								
	HEJ30	HEJ50	HEJ70	HES30	HES50	HES70	AMP^∗^	CHL^∗^	LEV^∗^
*Staphylococcus aureus* (ATCC® 6538)	>5000	>5000	5000	>5000	>5000	5000	<4	8	ND
*Staphylococcus aureus* (ATCC® 29213)	>5000	>5000	>5000	>5000	>5000	>5000	<4	8	ND
*Escherichia coli* (ATCC® 10536)	>5000	>5000	>5000	>5000	>5000	>5000	<4	<4	ND
*Escherichia coli* (ATCC® 25922)	5000	5000	5000	>5000	>5000	5000	<4	<4	ND
*Salmonella* Choleraesuis (ATCC® 10708)	5000	5000	5000	5000	5000	5000	<4	<4	<4
*Salmonella* Typhimurium (ATCC® 13311)	5000	5000	5000	5000	5000	5000	<4	<4	<4
*Pseudomonas aeruginosa* (ATCC® 9027)	>5000	>5000	>5000	>5000	>5000	>5000	>500	64	ND
*Pseudomonas aeruginosa* (ATTC® 27853)	>5000	>5000	>5000	>5000	>5000	>5000	>500	64	ND
Methicillin-resistant *Staphylococcus aureus* 1485279	5000	5000	5000	>5000	>5000	5000	500	125	ND
Methicillin-resistant *Staphylococcus aureus* 1605677	5000	5000	5000	5000	>5000	5000	125	8	ND
Methicillin-resistant *Staphylococcus aureus* 1664534	5000	5000	5000	>5000	>5000	5000	32	8	ND
Methicillin-resistant *Staphylococcus aureus* 1688441	>5000	>5000	>5000	>5000	>5000	>5000	250	250	ND
Methicillin-resistant *Staphylococcus aureus* 1830466	5000	5000	5000	5000	>5000	5000	125	<4	ND
*Salmonella Enteritidis* 1406591	5000	5000	5000	5000	5000	5000	<4	<4	<4
*Salmonella Enteritidis* 1418594	5000	5000	5000	5000	5000	5000	<4	<4	<4
*Salmonella Enteritidis* 1628260	5000	5000	5000	5000	5000	5000	<4	<4	<4
*Salmonella* spp. 1266695	5000	5000	5000	5000	5000	5000	<4	<4	<4
*Salmonella* spp. 1507708	5000	5000	5000	5000	5000	5000	>500	<4	8

ND: not determined. ^∗^MIC values of AMP, CHL, and LEV were in accordance with the ranges reported by the Clinical and Laboratory Standards Institute, document M100-S24 [31], classifying these bacteria as sensitive, intermediate, and resistant when appropriate. Experiments were carried out in quadruplicate and triplicate for the hydroethanolic extracts and for antibiotics, respectively.

## Data Availability

The data used to support the findings of this study are included within the article.

## Abbreviations

AMP: Ampicillin
AMR: Antimicrobial resistance
ATCC: American Type Culture Collection
BSI: Blood stream infection
CDC: Centers for Disease Control and Prevention
CHL: Chloramphenicol
DPPH: 2,2-Diphenyl-1-picryl-hydrazyl
GC: Growth control
HEJ30: Hydroethanolic extract of *Kalanchoe brasiliensis* leaves at 30% collected in January
HEJ50: Hydroethanolic extract of *Kalanchoe brasiliensis* leaves at 50% collected in January
HEJ70: Hydroethanolic extract of *Kalanchoe brasiliensis* leaves at 70% collected in January
HES30: Hydroethanolic extract of *Kalanchoe brasiliensis* leaves at 30% collected in September
HES50: Hydroethanolic extract of *Kalanchoe brasiliensis* leaves at 50% collected in September
HES70: Hydroethanolic extract of *Kalanchoe brasiliensis* leaves at 70% collected in September
HPLC-DAD: High-performance liquid chromatography with diode-array detection
LEV: Levofloxacin
MDR: Multidrug-resistant
MHA: Müeller-Hinton agar
MHB: Müeller-Hinton broth
MIC: Minimal inhibitory concentration
MRSA: Methicillin-resistant *Staphylococcus aureus*
MSSA: Methicillin-sensitive *Staphylococcus aureus*
ROS: Reactive oxygen species
TTC: 2,3,5-Triphenyl tetrazolium chloride
UHPLC-MS: Ultra-high-performance liquid chromatography coupled to a mass spectrometer
VISA: Vancomycin-intermediate *Staphylococcus aureus*
hVISA: Heteroresistant vancomycin-intermediate *Staphylococcus aureus.*

## Additional Points

*Highlights*. The hydroethanolic extracts from *K. brasiliensis* fresh leaves possess flavonoids (flavonols and flavones), mainly patuletin and eupafolin derivatives. HEJ70 and HES70 were the most active against the tested Gram-positive and Gram-negative bacterial strains. HEJ70 and HES70 were able to reduce the *Salmonella* growth rate. The mode of action of HEJ70 and HES70 on *Salmonella* probably involves the cellular membrane damage.

## Supplementary Materials

Supplementary Materials SUPP 1: Figure 1: UV-Vis spectra analysis of the six peaks detected in the chromatographic profiles of the 30%, 50%, and 70% hydroethanolic extracts of *Kalanchoe brasiliensis* fresh leaves collected in January (HEJ30, HEJ50, and HEJ70) by HPLC-DAD at 254 nm. UV spectrum of peaks 1 (A) flavonol indicating a methylated or glycosylated patuletin, 2 (B) flavonol indicating a methylated or glycosylated patuletin, 3 (C) flavone suggesting eupafolin or derivative or flavonol indicating a methylated or glycosylated patuletin, 4 (D) flavone suggesting eupafolin or derivative, 5 (E) flavonol indicating a methylated or glycosylated patuletin, and 6 (F) flavone suggesting eupafolin or derivative; flavonols with a substituted 3-hydroxyl group (methylated or glycosylated) can show band I at 328-357 nm. SUPP 2: Figure 2: UV-Vis spectra analysis of the six peaks detected in the chromatographic profiles of 30%, 50%, and 70% hydroethanolic extracts of *Kalanchoe brasiliensis* fresh leaves collected in September (HES30, HES50, and HES70) by HPLC-DAD at 254 nm. UV spectrum of peaks 1 (A) flavonol indicating a methylated or glycosylated patuletin, 2 (B) flavonol indicating a methylated or glycosylated patuletin, 3 (C) flavonol indicating a methylated or glycosylated patuletin, 4 (D) flavonol indicating a methylated or glycosylated patuletin, 5 (E) flavonol indicating a methylated or glycosylated patuletin, 6 (F) flavone suggesting eupafolin or derivative or flavonol indicating a methylated or glycosylated patuletin, and 7 (G) flavone suggesting eupafolin or derivative; flavonols with a substituted 3-hydroxyl group (methylated or glycosylated) can show band I at 328-357 nm.
