# Supplementary material for: Kalanchoe brasiliensis Cambess., a Promising Natural Source of Antioxidant and Antibiotic Agents against Multidrug-Resistant Pathogens for the Treatment of Salmonella Gastroenteritis
Source: Oxid Med Cell Longev. 2019 Nov 11;2019:9245951. doi: 10.1155/2019/9245951 (PMC6885303; doi:10.1155/2019/9245951)
Supplement: Supplementary Materials — SUPP 1: Figure 1: UV-Vis spectra analysis of the six peaks detected in the chromatographic profiles of the 30%, 50%, and 70% hydroethanolic extracts of Kalanchoe brasiliensis fresh leaves collected in January (HEJ30, HEJ50, and HEJ70) by HPLC-DAD at 254 nm. UV spectrum of peaks 1 (A) flavonol indicating a methylated or glycosylated patuletin, 2 (B) flavonol indicating a methylated or glycosylated patuletin, 3 (C) flavone suggesting eupafolin or derivative or flavonol indicating a methylated or glycosylated patuletin, 4 (D) flavone suggesting eupafolin or derivative, 5 (E) flavonol indicating a methylated or glycosylated patuletin, and 6 (F) flavone suggesting eupafolin or derivative; flavonols with a substituted 3-hydroxyl group (methylated or glycosylated) can show band I at 328-357 nm. SUPP 2: Figure 2: UV-Vis spectra analysis of the six peaks detected in the chromatographic profiles of 30%, 50%, and 70% hydroethanolic extracts of Kalanchoe brasiliensis fresh leaves collected in September (HES30, HES50, and HES70) by HPLC-DAD at 254 nm. UV spectrum of peaks 1 (A) flavonol indicating a methylated or glycosylated patuletin, 2 (B) flavonol indicating a methylated or glycosylated patuletin, 3 (C) flavonol indicating a methylated or glycosylated patuletin, 4 (D) flavonol indicating a methylated or glycosylated patuletin, 5 (E) flavonol indicating a methylated or glycosylated patuletin, 6 (F) flavone suggesting eupafolin or derivative or flavonol indicating a methylated or glycosylated patuletin, and 7 (G) flavone suggesting eupafolin or derivative; flavonols with a substituted 3-hydroxyl group (methylated or glycosylated) can show band I at 328-357 nm. [file 9245951.f1.docx]

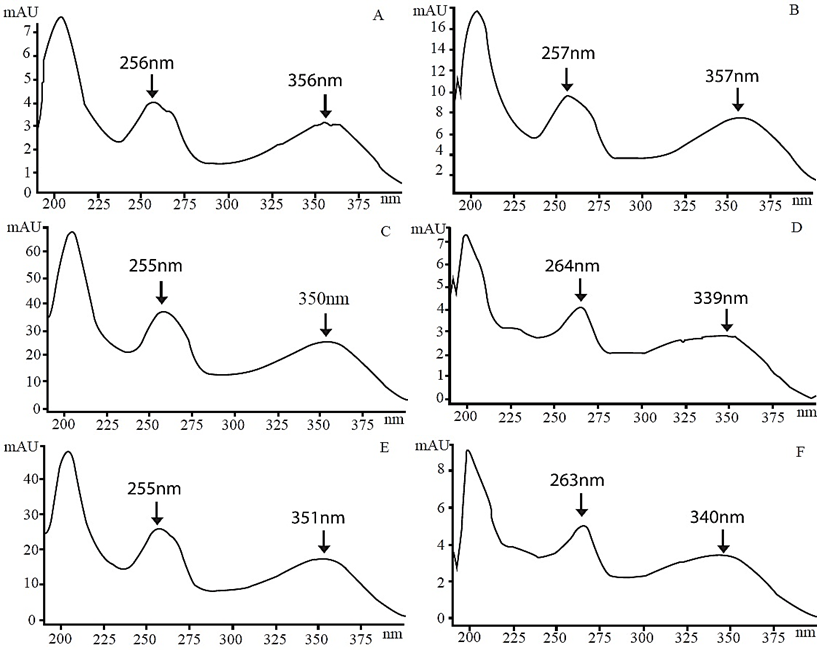


Figure 1: UV-Vis spectra analysis of the six peaks detected in the chromatographic profiles of the 30%, 50% and 70% hydroethanolic extracts of *Kalanchoe brasiliensis* fresh leaves collected in January (HEJ30, HEJ50 and HEJ70) by HPLC-DAD at 254 nm. UV spectrum of peaks 1 (A) - flavonol indicating a methylated or glycosylated patuletin, 2 (B) - flavonol indicating a methylated or glycosylated patuletin, 3 (C) - flavone suggesting eupafolin or derivative, or flavonol indicating a methylated or glycosylated patuletin, 4 (D) - flavone suggesting eupafolin or derivative, 5 (E) - flavonol indicating a methylated or glycosylated patuletin, and 6 (F) - flavone suggesting eupafolin or derivative; flavonols with a substituted 3-hydroxyl group (methylated or glycosylated) can show band I at 328 - 357 nm.


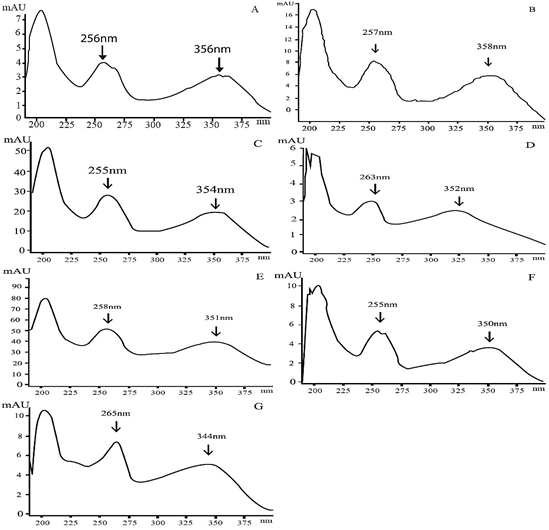


Figure 2: UV-Vis spectra analysis of the six peaks detected in the chromatographic profiles of 30%, 50% and 70% hydroethanolic extracts of *Kalanchoe brasiliensis* fresh leaves collected in September (HES30, HES50 and HES70) by HPLC-DAD at 254 nm. UV spectrum of peaks 1 (A) - flavonol indicating a methylated or glycosylated patuletin , 2 (B) - flavonol indicating a methylated or glycosylated patuletin, 3 (C) - flavonol indicating a methylated or glycosylated patuletin, 4 (D) - flavonol indicating a methylated or glycosylated patuletin, 5 (E) - flavonol indicating a methylated or glycosylated patuletin, 6 (F) - flavone suggesting eupafolin or derivative, or flavonol indicating a methylated or glycosylated patuletin , and 7 (G) - flavone suggesting eupafolin or derivative; flavonols with a substituted 3-hydroxyl group (methylated or glycosylated) can show band I at 328 - 357 nm.
